# Rivastigmine for minor visual hallucinations in Parkinson's disease: A randomized controlled trial with 24 months follow‐up

**DOI:** 10.1002/brb3.2257

**Published:** 2021-07-21

**Authors:** Tom J. M. van Mierlo, Elisabeth M. J. Foncke, Bart Post, Ben A. Schmand, Bastiaan R. Bloem, Barbera van Harten, Gerrit Tissingh, Alexander G. Munts, Rob J. de Haan, Rob M. A. de Bie, other individuals of the CHEVAL Study Group, Pieter Nederveen, Jean Michel Krul, Annemarie Vlaar, Agnita Boon, Henk Berendse, Jons Verduijn, Mirthe Ponssen, Teun van Strien, Nathalie Rosenberg, Jorrit Hoff, Bob van Hilten, Roeland Tans, Germ Tiessens, Annemarie Wijnhoud, Willem Van der Meulen, Dareia Roos, Jeroen van Vugt, Ties van Asseldonk, Gerwin Roks, Chris Fokke, Erik van Wensen, Ad Vermeij, Lucile Dorresteijn, Sarah Vermeer, Mark Kuijf, Teus van Laar, Mirjam van Kesteren, Niek Verweij, Wouter Schuiling, Axel Portman, Philip Scheltens, Koos Zwinderman, Joke Dijk, Michel Hof

**Affiliations:** ^1^ Department of Neurology Amsterdam Neuroscience, VU University Amsterdam Amsterdam University Medical Centers Amsterdam The Netherlands; ^2^ Department of Neurology and Centre of Expertise for Parkinson and Movement Disorders Donders Institute for Brain Cognition and Behaviour, Radboudumc Nijmegen The Netherlands; ^3^ Department of Psychology University of Amsterdam Amsterdam The Netherlands; ^4^ Department of Neurology Medical Center Leeuwarden Leeuwarden The Netherlands; ^5^ Department of Neurology Zuyderland Medical Center Heerlen The Netherlands; ^6^ Department of Neurology Spaarne Gasthuis Haarlem The Netherlands; ^7^ Clinical Research Unit Amsterdam Neuroscience, University of Amsterdam Amsterdam University Medical Centers Amsterdam The Netherlands; ^8^ Department of Neurology Amsterdam Neuroscience, University of Amsterdam Amsterdam University Medical Centers Amsterdam The Netherlands; ^9^ Dijklander Hospital Hoorn The Netherlands; ^10^ Ter Gooi Hospital Blaricum The Netherlands; ^11^ Onze Lieve Vrouwe Gasthuis Amsterdam The Netherlands; ^12^ Erasmus Medical Center Rotterdam The Netherlands; ^13^ Amsterdam University Medicale Centers location Boelelaan Amsterdam The Netherlands; ^14^ Flevo Hospital Almere The Netherlands; ^15^ Meander Medical Center Amersfoort The Netherlands; ^16^ Bravis Hospital Roosendaal The Netherlands; ^17^ St. Antonius Hospital Nieuwegein The Netherlands; ^18^ Leiden University Medical Center Leiden The Netherlands; ^19^ ACIBADEM International Medical Center Amsterdam The Netherlands; ^20^ Ijsselland Hopsital Cappelle aan de Ijssel The Netherlands; ^21^ Rode Kruis Hospital Beverwijk The Netherlands; ^22^ Medisch Spectrum Twente Enschede The Netherlands; ^23^ Elisabeth Twee Steden Hospital Tilburg The Netherlands; ^24^ Gelre Hospital Apeldoorn The Netherlands; ^25^ Catharina Hospital Eindhoven The Netherlands; ^26^ Rijnstate Hospital Arnhem The Netherlands; ^27^ Maastricht University Medical Center Maastricht The Netherlands; ^28^ Univesity Medical Center Groningen Groningen The Netherlands; ^29^ Isala Hospital Zwolle The Netherlands; ^30^ Medical Center Leeuwarden Leeuwarden The Netherlands; ^31^ Refaja Hospital Stadskanaal The Netherlands; ^32^ Amsterdam University Medicale Centers location AMC Amsterdam The Netherlands

**Keywords:** cholinesterase inhibitors, hallucinations, Parkinson's disease, psychosis, randomized controlled trial

## Abstract

**Background:**

Visual hallucinations are common in patients with Parkinson's disease and represent probably the major independent predictor for cognitive deterioration and nursing home placement.

**Objective:**

To investigate if treatment of minor visual hallucinations in Parkinson's disease with rivastigmine delays the progression to psychosis.

**Methods:**

A multicenter, randomized, double‐blind, placebo‐controlled trial was conducted which aimed to recruit 168 patients with Parkinson's disease reporting minor visual hallucinations 4 weeks before it. Important exclusion criteria were Parkinson's disease dementia, current delirium, and treatment with antipsychotics or drugs that have significant anti‐cholinergic side effects. Subjects were randomized to rivastigmine capsules, 3–6 mg twice a day, or placebo for 24 months. The primary outcome was the time to Parkinson's disease psychosis, which was defined as the need to start with antipsychotics.

**Results:**

The trial was stopped prematurely because of slow recruitment. Ninety‐one patients were randomized: 46 patients were assigned to rivastigmine and 45 patients to placebo. No effect of rivastigmine could be demonstrated on the transition time to psychosis or dementia during the 24‐month follow‐up period. After 6 months of study treatment, cognition, mood, motor performance, and non‐motor performance did not differ significantly between the rivastigmine‐group and the placebo‐group.

**Conclusions:**

Because the study was terminated early, it was insufficiently powered to properly evaluate the primary outcome. The limited data of the study favor a wait and see approach instead of early treatment with rivastigmine in PD patients with minor VH.

## Graphical abstract

A randomized controlled trial was conducted to investigate if treatment of minor visual hallucinations in Parkinson's disease with rivastigmine delays the progression to psychosis. The limited data of our study favor a wait and see approach instead of early treatment.

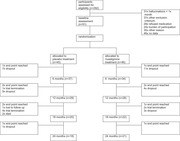

## 1 INTRODUCTION

Visual hallucinations (VH) are common in patients with Parkinson's disease (PD) and represent probably the major independent predictor for cognitive deterioration and nursing home placement (Diederich et al., 2009; Kempster et al., 2010). Many patients present with minor VH, including presence or passage phenomena, which over time often progress to major VH with well‐formed images and psychosis with delusions, where there is loss of insight into the false nature of these hallucinations and beliefs (Goetz et al., 2006).

Retrospective data suggest that early treatment of minor VH with clozapine can delay the transition to psychosis (Goetz et al., 2008). However, many regard clozapine unsuitable for long‐term use in PD because of the risk for serious side effects, such as agranulocytosis, orthostatic hypotension, and sedation (Wang et al., 2005; The Parkinson Study Group, 1999).

Cholinesterase inhibitors are a recommended treatment of PD dementia and might be a potential alternative to clozapine for the treatment of VH (Seppi et al., 2019). A large randomized controlled trial of rivastigmine demonstrated that both cognition and behavior were improved at a group level in patients with PD dementia (Emre et al., 2004). Furthermore, a post hoc analysis reported a greater therapeutic benefit for those PD dementia patients who also experience VH (Burn et al., 2006).

We initiated a clinical trial to investigate whether the treatment of minor VH with rivastigmine delays the progression to PD psychosis. In addition, we anticipated to confirm an immediate effect on the severity of VH. Unfortunately, the trial had to be stopped prematurely, because of slow recruitment. We present the results of the trial according to an adapted version of the original statistical analysis plan.

## 2 METHODS

### 2.1 Trial overview

We conducted a multicenter, randomized, double‐blind, placebo‐controlled trial. The ethics committee of the Amsterdam University Medical Centers in the Netherlands approved the protocol. The trial was conducted in accordance with the principles of the Declaration of Helsinki. The trial was registered on http://www.clinicaltrials.gov (NCT01856738). Trial monitoring and data management were performed in accordance with the International Conference on Harmonisation Good Clinical Practice guidelines. All the patients provided written informed consent. An independent Data and Safety Monitoring Board (DSMB) monitored safety and efficacy data.

### 2.2 Patients

The inclusion criteria were PD according to diagnostic criteria (Hughes et al., 1992) in the last 4 weeks minor VH defined by a score of 1 or 2 on the hallucinations item of the Movement Disorders Society Unified Parkinson's Disease rating Scale (MDS‐UPDRS; Goetz et al., 2008) and age 40 years and above. Important exclusion criteria were delirium, PD psychosis (defined as treatment with antipsychotic drugs), PD dementia (defined as MMSE‐score < 26 at baseline), treatment with cholinesterase inhibitors in the past 6 months, and VH that appeared within 1 month following the increase of dopaminergic treatment. We also excluded subjects taking drugs with significant anticholinergic side effects (including amantadine), subjects with a history of PD psychosis and subjects who stayed permanently in a nursing home. Subjects were recruited from 30 community hospitals and 7 academic hospitals in the Netherlands.

### 2.3 Trial procedures

After the baseline assessment (visit 1), subjects were randomized using a centralized web‐based application in a 1:1 ratio to receive rivastigmine (rivastigmine‐group) or placebo (placebo‐group). Randomization was stratified by type of hospital (University Medical Center versus non‐University Medical Center) and age (below 65 years or 65 years and older) using variable permuted blocks. Study personnel, research nurses, neurologists, and subjects were blinded to the treatment allocation until the database was locked.

Rivastigmine dosage was increased according to a standardized titration schedule in the first 12 weeks from 1.5 mg capsules to the maintenance dose of 6.0 mg capsules BID. Masking was achieved with matched placebo capsules and a dummy up titration schedule. In case of persisting side effects, the dose was lowered. The subject was instructed to keep the highest tolerated dose until the end of the study with a minimum of rivastigmine 3.0 mg BID. The treatment started as soon as possible after randomization (with a maximum of 6 weeks after visit 1).

Four specified follow‐up visits (at home or at the outpatient clinic) were planned at 6, 12, 18, and 24 months after the start of treatment (visits 2, 3, 4, and 5, respectively). In addition, subjects were asked about side effects in a telephone interview during the titration period (after 2, 3, 5, 8, and 11 weeks of treatment).

If a subject developed PD psychosis (primary endpoint), the study medication was discontinued and an extra visit was scheduled within 6 weeks if the subject was still able to perform tests. Planned visits, except the last visit (visit 5), were cancelled after the primary endpoint was reached.

If a subject met the criteria for PD dementia (secondary endpoint), the subject was instructed to stop taking the study medication. Subsequently, rivastigmine was prescribed by the treating neurologist according to the current guideline (Rogers et al., 2017). This secondary endpoint was added after the trial started because subjects turned out to convert to dementia before converting to psychosis and rivastigmine is considered the standard care for PD dementia.

### 2.4 Outcome measures

The primary outcome was the time to PD psychosis observed during the 24‐months follow‐up period. PD psychosis was defined as a behavior that required the use of antipsychotics according to the treating neurologist. Secondary outcomes included the time to PD dementia, diagnosed by the treating neurologist according to current guidelines (disability in > 1 cognitive domain and a score < 26 on the Mini Mental State Examination; Emre et al., 2007). Other secondary outcomes, assessed at 6‐month, were: severity of PD symptoms (MDS‐UPDRS part 1–3; Goetz et al., 2008) psychotic symptoms (Scale to Assess Positive Symptoms; Andreasen, 1990) cognitive function (Montreal Cognitive Assessment; Parkinson's Disease Cognitive Rating Scale; Nasreddine et al., 2005; Pagonabarraga et al., 2008) mood (Hospital Anxiety and Depression Scale; Zigmond & Snaith, 1983) daytime sleepiness (Epworth Sleepiness Scale; Johns, 1991) disability (AMC Linear Disability Score; Weisscher et al., 2007) adverse events, and compliance to study treatment.

### 2.5 Sample size calculation

Based on two retrospective studies that investigated early treatment of VH with clozapine and presuming an exponential disease course we assumed that within the 24‐month follow‐up period 35% of the subjects in the placebo‐group would not reach the primary endpoint (PD psychosis) versus 60% in the rivastigmine‐group (Goetz et al., 2006; Goetz et al., 2008). With a sample size in each group of 63 subjects (126 in total), a 0.05 level two‐sided log‐rank test for equality of survival curves would have 80% power to detect the difference between the placebo‐group proportion at 24 months of 0.35 (proportion of subjects without psychosis) and the rivastigmine‐group proportion at 24 months of 0.60 (proportion of subjects without psychosis), with a constant hazard ratio of 2.055. Anticipating a dropout rate of 25%, we needed 63/0.75 = 84 subjects per treatment group (168 in total).

### 2.6 Statistical analysis

Analyses were based on the intention‐to‐treat principle. Baseline characteristics and outcome parameters, including adverse events, were summarized using descriptive statistics. The main analysis of this trial consisted of a univariate comparison between the treatment groups for the primary outcome within a 24‐month follow‐up period. The between‐group difference in time until subjects progressed to PD psychosis was analyzed by plotting Kaplan–Meier curves and comparing them using the log‐rank test. The same statistical approach was used with regard to the between‐group difference in time to PD dementia (secondary outcome). With regard to the remaining secondary outcomes on cognition and behavior (MDS‐UPDRS part 1–3, SAPS, MoCA, PD‐CRS, HADS, ESS, ALDS) we compared the change scores (from baseline to 6‐month follow‐up) between treatment groups, using the Pearson's Chi‐squared (*χ* ^2^) test, Welch's two sample *t*‐test, and Wilcoxon rank sum test (with continuity correction), where appropriate. A two‐sided *p* value less than .05 was considered statistically significant. We did not correct for multiple testing.

## 3 RESULTS

### 3.1 Subjects

**FIGURE 1:**
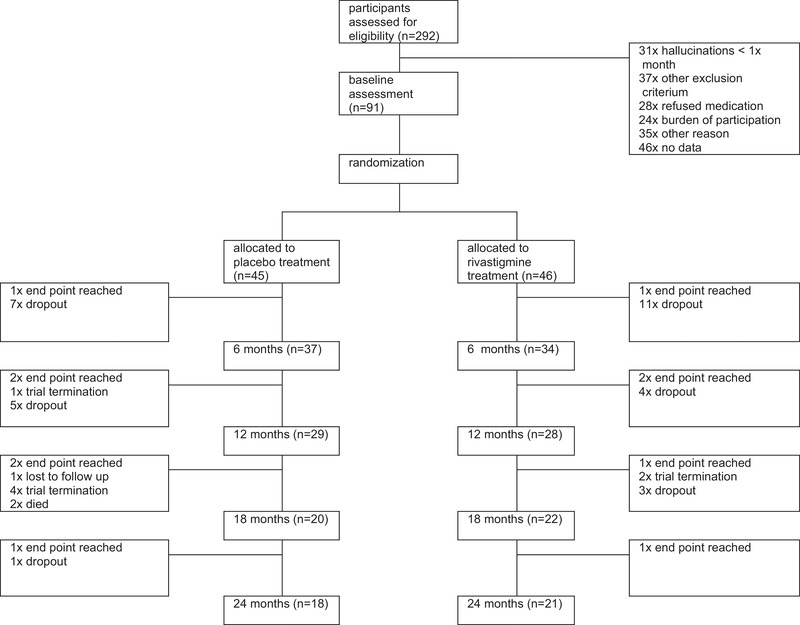
Enrollment and randomization

From November 2013 through December 2017, 292 subjects were screened for participation in the study. Ninety‐one subjects were enrolled and randomized; 46 subjects were assigned to rivastigmine and 45 to placebo (Figure 1). Baseline demographic and clinical characteristics of the two groups were comparable (Table 1). From the start of the trial, recruitment was slow despite numerous efforts to increase the inclusion rate. After an inclusion period of three‐and‐a‐half years, the DSMB performed an interim futility analysis on the primary outcome with a total of 80 included patients (39 assigned to rivastigmine and 41 to placebo). The DSMB concluded that the trial did not seem to be futile and advised to continue the study. Despite new strategies, the inclusion rates again had not improved during the six months after the interim analysis. Eventually the study group decided that it was no longer feasible to achieve the original study target. In consultation with the funding partners, the trial was ended prematurely. Thirty‐four patients in the rivastigmine‐group and 37 patients in the placebo‐group completed the 6‐month follow‐up period. Twenty‐one patients in the rivastigmine‐group and 18 patients in the placebo‐group completed the 24‐month follow‐up period (Figure 1).

**TABLE 1:** Demographic and baseline clinical characteristics

Variables	Rivastigmine *N* = 46	Placebo *N* = 45
Mean age in years ± SD	66.9 ± 7.9	66.6 ± 8.0
Number aged 65 years or older (%)	29 (63%)	28 (62%)
Number of women (%)	14 (30%)	12 (27%)
Years since diagnosis ± SD	8.0 ± 5.2	5.9 ± 4.3
Years since onset of visual hallucinations ± SD	2.2 ± 2.8	2.3 ± 2.7
Number recruited from university hospital (%)	13 (28%)	12 (27%)
MDS‐UPDRS score^a^		
Mean part 1 ± SD	15.4 ± 6.7	14.2 ± 4.4
Mean part 2 ± SD	14.3 ± 7.9	14.2 ± 5.9
Mean part 3 ± SD	30.7 ± 17.1	34.9 ± 12.1
Median MMSE (range)^b^	29 (26–30)	29 (26–30)
Median MoCa (range)^c^	24 (19–30)	25 (16–30)
Median HADS (range)^d^	10 (0–33)	11 (0–42)

Abbreviations: HADS, Hamilton anxiety and depression Score; MDS‐UPDRS, movement disorders society unified Parkinson's disease rating scale; MMSE, mini‐mental sate examination; MoCa, Montreal Cognitive Assessment.

^a^ MDS‐UPDRS scores range from 0 to 176, with higher scores indicating more severe disease; the scale includes subscales of mental function (part 1, range 0–52), activities of daily living (part 2, range 0–52) and motor function (part 3, range 0–132).

^b^ MMSE scores range from 0 to 30, with lower scores indicating greater cognitive impairment.

^c^ MoCa scores range from 0 to 30, with lower scores indicating greater cognitive impairment.

^d^ HADS scores range from 0 to 42, with higher scores indicating more severe affective symptoms.

### 3.2 Outcomes

**FIGURE 2:**
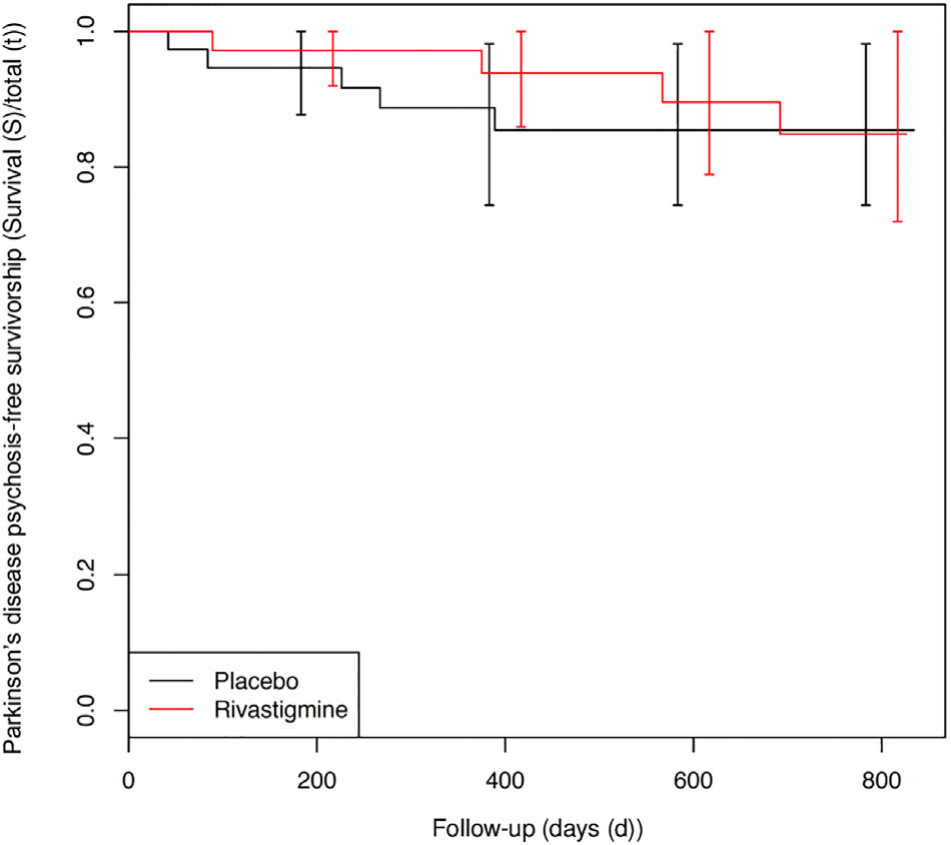
Primary outcome: time in days before visual hallucinations progress to Parkinson's disease psychosis (Kaplan–Meier curve)

Figure 2 presents the between‐group difference in time to PD psychosis (primary outcome). No significant difference between the survival curves could be demonstrated (*p* = .70; log‐rank test). In the rivastigmine‐group, 4 out of 46 subjects developed PD psychosis, and in the placebo‐group 5 out of 45 developed PD psychosis.

**FIGURE 3:**
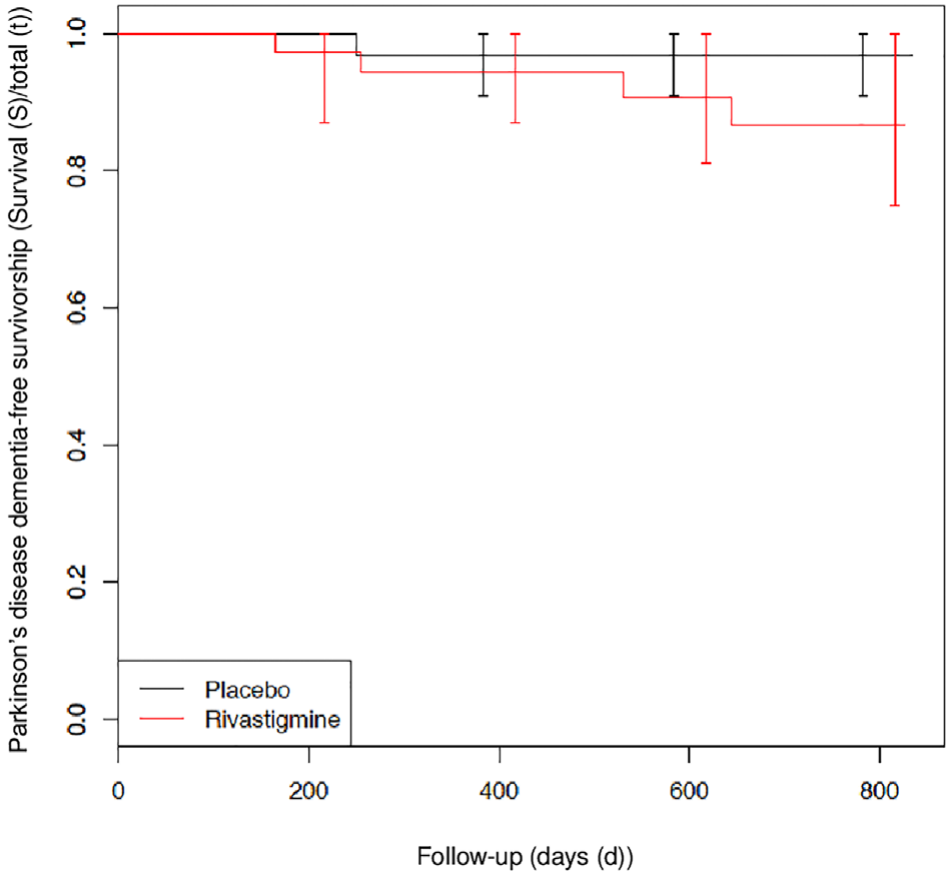
Secondary outcome: time in days before visual hallucinations progress to Parkinson's disease dementia (Kaplan–Meier curve)

Between‐group difference in time to PD dementia (secondary outcome) is depicted in Figure 3. No significant difference between the curves was observed (*p* = .20). In the rivastigmine‐group, 4 out of 46 subjects developed PD dementia and in the placebo‐group 1 out of 45 developed PD dementia.

**FIGURE 4:**
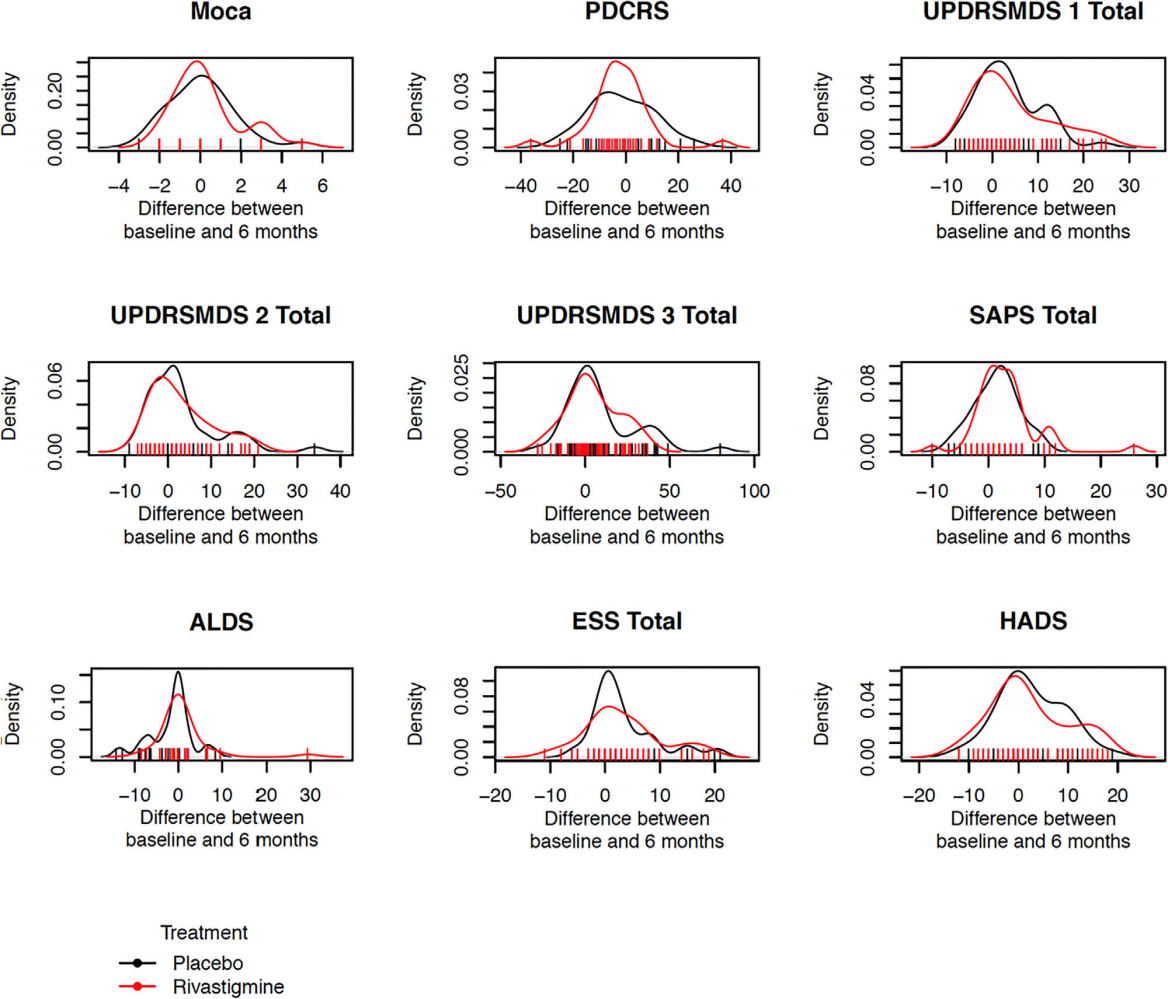
Secondary outcomes: differences in scores change after six months of treatment

After 6 months of treatment, there were no statistically significant differences in change scores between the treatment groups on the MDS‐UPDRS part 1 (*p* = 0.98; Wilcoxon rank sum test), MDS‐UPDRS part 2 (*p* = 0.71; Wilcoxon rank sum test), MDS‐UPDRS part 3 (*p* = 0.54; Wilcoxon rank sum test), SAPS (*p* = 0.13; Welch's *t*‐test); MoCA (*p* = 0.28; *χ* ^2^ test), PD‐CRS (*p* = 0.75; Welch's *t*‐test), HADS (*p* = 0.83; Welch's *t*‐test), ESS (*p* = 0.97 Welch's *t*‐test), and ALDS (*p* = 0.06; Welch's *t*‐test; Figure 4).

**FIGURE 5:**
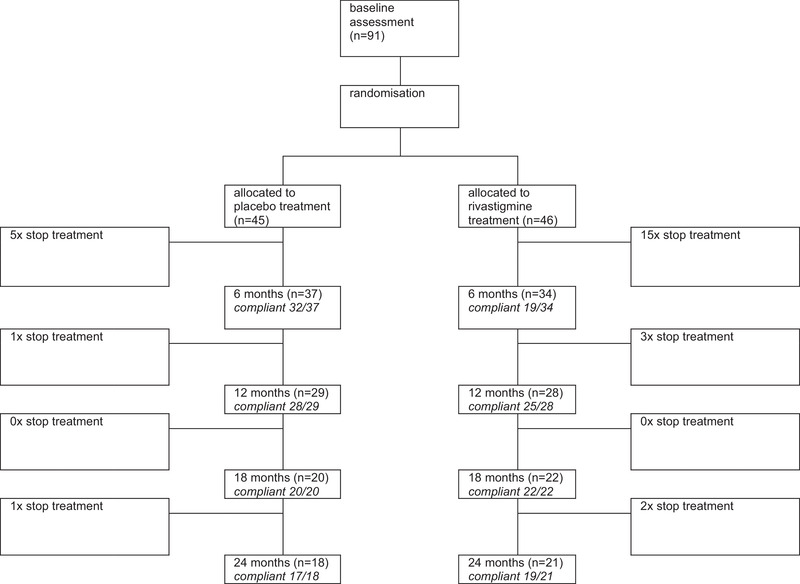
Compliance

The subjects in the rivastigmine‐group stopped study medication more often compared to the subjects in the placebo‐group (20 out of 46 and 7 out of 45, respectively). The majority of subjects stopped with the study medication within the first six months (15 of 20 and 5 of 7, respectively; Figure 5). Adverse events reported in the first three months formed a likely explanation. In the first 12 weeks, gastro‐intestinal symptoms (in particular nausea, vomiting, and diarrhea) seemed to occur more frequently in the rivastigmine‐group compared to the placebo‐group. Notably, nausea was reported more often during the early titration phase (week 3) in the placebo‐group.

## 4 DISCUSSION

We investigated the merits of early treatment of minor VH in PD with rivastigmine. Only few prior, single‐center trials studied rivastigmine in a non‐demented PD population and although the final sample size of our multicenter trial was relatively small and well below our predefined targets, it is one of the largest to date and with good external validity (Henderson et al., 2016; Mamikonyan et al., 2015). The trial was terminated prematurely due to slow recruitment. Therefore, the hypothesis that cholinesterase inhibitors, when given to patients with PD who experience VH, can delay the progression to PD psychosis could neither be confirmed nor firmly rejected. In addition, we could not demonstrate benefit from rivastigmine treatment after six months on any of the secondary outcome measures: (non)‐motor symptoms, psychotic symptoms, cognition, mood, daytime sleepiness, or disability. This agrees with previous research on the use of rivastigmine in patients with PD and mild cognitive impairment showing no advantage on behavioral symptoms (Mamikonyan et al., 2015). Taken together, this suggests that rivastigmine only improves behavioral outcomes in patients with established PD dementia (Emre et al., 2004).

In our study, the number of patients that converted from having minor VH to PD psychosis in our randomized controlled trial was rather low (9 out of 91 subjects converted in 2 years) when compared to the retrospective cohort‐study by Goetz and colleagues (39 out of 48 subjects converted in 3 years; Goetz et al., 2006), possibly because we included patients that experienced VH only once per month, and were not necessarily troubled by these hallucinations. Interestingly, 10% of the patients that were referred and screened for the trial could not participate because they had a VH frequency of less than one per month. However, it should be noticed that the frequency of the VH is difficult to determine: the experience is not only subjective, but often also brief and not always reported or remembered. In addition, an ongoing taboo to discuss hallucinations in the consulting room also may influence the real prevalence rate of VH. Taking this together, the amount of suitable candidates for recruitment in the present study was unfortunately rather low (Fénelon et al., 2000).

Slow recruitment can also be the result of a limited interest in the trial. First, patients do not consider their VH debilitating. Second, neurologists already prescribe rivastigmine off label to reduce troublesome VH in PD without dementia.

After appropriate counseling, potentially eligible subjects found the trial to be rather burdening. They opted out as they were concerned that drug treatment would worsen their overall performance. Furthermore, we wrongly assumed that PD patients would be convinced about the positive effect on quality of daily life of drug treatment. In the absence of a short‐term gain, the 24‐month follow‐up period might have been too intensive for this fragile target population.

As mentioned before, the dropout rates were high and treatment compliance was low. This might have affected the outcome negatively. Earlier reports provided different findings on rivastigmine treatment withdrawal and the number of (gastro‐intestinal) adverse events. Our trial is not the first to find a 40% discontinuation rate and around 30% gastro‐intestinal side effects approximately (Henderson et al., 2016). The total number of side effects in the rivastigmine‐group was higher than in the placebo‐group. The low tolerance for oral medication and consequently low dosages used may have been a further limiting factor that could explain why we found no significant benefits for rivastigmine treatment, not even on cognitive functioning. Transdermal administration of rivastigmine probably would have led to a lower dropout rate and higher mean maintenance dose (Emre et al., 2014). However, it is possible that patients require an obvious, immediate benefit to improve long‐term compliance, sufficiently to test for possible long‐term effects.

In summary, the limited data of our study favor a wait and see approach instead of early treatment with rivastigmine in PD patients with minor VH. Minor VH in PD patients are difficult to measure, are not always considered debilitating and progresses slowly. Compliance with rivastigmine as a preventive treatment is low. We did not find a reason to start rivastigmine treatment before the criteria for PD dementia are met. This is in accordance with the current guidelines (Connolly & Lang, 2014; Rogers et al., 2017; Seppi et al., 2019).

## CONFLICT OF INTEREST

The authors have no conflict of interest to report.

### PEER REVIEW

The peer review history for this article is available at https://publons.com/publon/10.1002/brb3.2257.

## OTHER INDIVIDUALS OF THE CHEVAL STUDY GROUP

**Table:** 

Name	Location
Pieter Nederveen, MD	Dijklander Hospital, Hoorn, The Netherlands
Jean Michel Krul, MD	Ter Gooi Hospital, Blaricum, The Netherlands
Annemarie Vlaar, MD, PhD	Onze Lieve Vrouwe Gasthuis, Amsterdam, The Netherlands
Agnita Boon, MD, PhD	Erasmus Medical Center, Rotterdam, The Netherlands
Henk Berendse, MD, PhD	Amsterdam University Medicale Centers location Boelelaan, Amsterdam, The Netherlands
Jons Verduijn, MD	Flevo Hospital, Almere, The Netherlands
Mirthe Ponssen, MD, PhD	Meander Medical Center, Amersfoort, The Netherlands
Teun van Strien, MD	Bravis Hospital, Roosendaal, The Netherlands
Nathalie Rosenberg, MD, PhD	Dijklander Hospital, Hoorn, The Netherlands
Jorrit Hoff, MD, PhD	St. Antonius Hospital, Nieuwegein, The Netherlands
Bob van Hilten, MD, PhD	Leiden University Medical Center, Leiden, The Netherlands
Roeland Tans, MD	ACIBADEM International Medical Center, Amsterdam, The Netherlands
Germ Tiessens, MD	Onze Lieve Vrouwe Gasthuis, Amsterdam, The Netherlands
Annemarie Wijnhoud, MD, PhD	Ijsselland Hopsital, Cappelle aan de Ijssel, The Netherlands
Willem Van der Meulen, MD	Rode Kruis Hospital, Beverwijk, The Netherlands
Dareia Roos, MD	Amsterdam University Medicale Centers location Boelelaan, Amsterdam, The Netherlands
Jeroen van Vugt, MD, PhD	Medisch Spectrum Twente, Enschede, The Netherlands
Ties van Asseldonk, MD, PhD	Elisabeth Twee Steden Hospital, Tilburg, The Netherlands
Gerwin Roks, MD, PhD	Elisabeth Twee Steden Hospital, Tilburg, The Netherlands
Chris Fokke, MD	Gelre Hospital, Apeldoorn, The Netherlands
Erik van Wensen, MD, PhD	Gelre Hospital, Apeldoorn, The Netherlands
Ad Vermeij, MD	Catharina Hospital, Eindhoven, The Netherlands
Lucile Dorresteijn, MD, PhD	Medisch Spectrum Twente, Enschede, The Netherlands
Sarah Vermeer, MD, PhD	Rijnstate Hospital, Arnhem, The Netherlands
Mark Kuijf, MD, PhD	Maastricht University Medical Center, Maastricht, The Netherlands
Teus van Laar, MD, PhD	Univesity Medical Center Groningen, Groningen, The Netherlands
Mirjam van Kesteren, MD	Isala Hospital, Zwolle, The Netherlands
Niek Verweij, MD, PhD	Medical Center Leeuwarden, Leeuwarden, The Netherlands
Wouter Schuiling, MD	Medical Center Leeuwarden, Leeuwarden, The Netherlands
Axel Portman, MD, PhD	Refaja Hospital, Stadskanaal, The Netherlands
Philip Scheltens, MD, PhD	Amsterdam University Medicale Centers location Boelelaan, Amsterdam, The Netherlands
Koos Zwinderman, MD, PhD	Amsterdam University Medicale Centers location AMC, Amsterdam, The Netherlands
Joke Dijk, MD, PhD	Amsterdam University Medicale Centers location AMC, Amsterdam, The Netherlands
Michel Hof, PhD	Amsterdam University Medicale Centers location AMC, Amsterdam, The Netherlands

## Data Availability

Anonymized data will be shared by request from any qualified investigator. Data will be available beginning 12 months and ending 5 years following the publication of the article. Requests for access to data must be accompanied by a methodologically sound proposal. Requests can be addressed to t.vanmierlo@amsterdamumc.nl . A signed data sharing agreement is required before access can be provided. The study protocol and statistical analysis plan are available online (www.chevalstudie.nl).
